# Analytical Review of Select Hospital Reports in the Lancet

**Published:** 1827-07

**Authors:** 


### ANALYTICAL REVIEW OF SELECT HOSPITAL REPORTS IN THE LANCET.

In this Review, as in the analysis of other journals, foreign and domestic, we shall greatly abridge and condense the original reports, by avoiding all circumlocution and unnecessary details. A majority of the cases we shall pass entirely unnoticed, as devoid of interest or practical information. In our comments, we shall be guided by rigid impartiality and a sincere desire to promulgate truth and utility.

### I.

### BARTHOLOMEW'S HOSPITAL.

1. *Fatal Phlebitis. Case* 1. [Lancet, No. 183.] ———, ætat. 25, who had married at the age of 15, and been in the habit of drinking spirits freely from that time, was bled in the left median basilic vein, for some bruises she had received. Next day she returned to her usual occupation of weaving, (qy. drinking?) but in the evening the arm felt stiff and painful, and the bandage tight. The symptoms got worse, and Dec. 2, the 5th day from the phlebotomy, she entered Bartholomew's Hospital. The arm for some distance above and below the inside of the elbow joint was swollen, hard, red, and very painful on pressure, and there was a small crust on the wound in the vein. The axillary glands were unaffected. There was considerable constitutional irritation, and 16 ounces of blood were taken from the other arm, which made her faint. "Blood highly cupped and buffed; twenty leeches to the inflamed arm in the evening; calomel and jalap; and the saline medicine, with antimony every six hours." Dec. 3d. Pain in the abdomen, increased on pressure, or a deep inspiration; in other respects rather worse. Thirty leeches to the inflamed arm. 4th. Epigastrium very tender; thirty leeches to it. 5th. Arm rather easier, but the inflammation is spreading to the axilla; pain in epigastrio gone.—*Calomel,* gr. iij. 4*tis horis.—Saline to be discontinued.* 7th. Wound in the arm discharges freely pus, sometimes mixed with blood, a considerable quantity of which issues from the vein on pressing the neighbouring parts. Pulse small, and 104—tongue furred—severe pains all over the body, particularly in the extremities. 8th. Much the same; the calomel, apparently beginning to affect the mouth, is to be discontinued—*salines*—twenty-five leeches to the fore-arm—fomentations. 10th. Much better—swelling and pain of the arm nearly gone, but thin pus still exudes from the orifice of the vein. On the morning of the 11th she seemed better, but in the evening she became pale and anxious; the skin was cold, the respiration 30 in the minute and laborious; pulse 104 and weak—abdomen painful on pressure—purging. 12th. Matter having formed under the skin of the *right* arm, without redness, a puncture was made, and five ounces of good pus evacuated. "Painful swelling from effusion into the articular cavity." Ten grains of calomel and one of opium. 13th. The calomel has caused violent purging, and she is now evidently sinking. 14th. Died this morning.

*Sectio Cadaveris.* The husband was present, and the examination consequently hurried. There was inflammatory condensation in the arm and fore-arm; a chain of small suppurations, in the inflamed parts, along the course of the blood-vessels, with healthy pus from the elbow to the axilla. Axillary and subclavian veins, with the upper cava and heart natural. No disease in the chest nor abdomen, save that the liver was taking on the yellow appearance induced by indulgence in spirits.

*Remarks.* This case exemplifies well the melancholy manner in which the lower orders in London destroy their constitutions, not very good, perhaps, at the best, by the immoderate use of spirits. Here we see a wretched woman beginning to drink at the age of 15! and we cannot be surprised that, after ten years' habitual intoxication, she had not stamina enough to support so simple an operation as that of phlebotomy.

*Case* 2.* H. A. having an inflamed ulcer on the leg, was bled at St. Bartholomew's on the 25th of January. On the 28th he felt some stiffness in the elbow-joint, (the left,) and during the night his sleep was broken, whilst he felt a peculiar sensation about his head. Next night he was restless and rather delirious. 30th. The arm was hot, tense, and much swollen, the tumefaction extending beyond the lower margin of the pectoralis major; the veins were enlarged and cord-like; the axillary glands enlarged also, and the absorbents traceable into them. There was considerable constitutional excitement. V.S. ad ℥xvi.—calomel and jalap immediately—and one grain of the antimonium tartarizatum, with half a drachm of potassæ nitras, in solution, every four hours. 31st. Much the same; the blood was not buffed nor cupped—thirty leeches were ordered to the arm, and vesication by a large blistering plaster. The antimony had been discontinued by the dresser, from its producing nausea. *Feb.* 1st. Great constitutional disturbance, the symptoms taking on more of a typhoid character. "The actual state of the limb could not be ascertained, on account of the irritating effects of the blister." Mr. Lawrence ordered the antimony to be resumed. 2d. Rather worse—symptoms of cerebral disturbance. 3d. The antimony, having caused vomiting and nausea, is to be left off. Twelve minims of tincture of digitalis in ℥ss. of saline mixture every four hours. 4th. Pulse 130—severe darting pains in the thigh and calf of the left leg. 5th. He is decidedly better. On turning down the bed-clothes to look at the ulcer on the leg, the *right* knee was found to be much swollen, tense, hard, and unfluctuating;" the thigh was tumefied, and the superficial veins much injected. Cucurb. cruent. ad ℥xvi.—fomentations—"four grains of calomel in a pill, with the digitalis mixture as before, with an addition of three drops of the tinct. digit. made every four hours." 6th. He is better to-day; some pain in the right shoulder. 7th. The tongue is brown and parched—the countenance cadaverous—pulse 116—motions passed involuntarily. The thigh more swollen and livid. In the evening he became comatose, and at half-past four next morning he died.

*Sectio Cadaveris.* On removing the integuments, a small quantity of pus was seen pointing at the aperture of the median basilic vein, the coats of

* Lancet, No. 184.

which were thickened to about midway up the arm. "The vein was pervious throughout." On cutting into the knee-joint, pus and blood flowed from the opening. There was ulceration of the cartilages, the surrounding unabsorbed portion being minutely injected with blood. On making an incision above the joint, the cellular tissue between the rectus and cruræus was found infiltrated with healthy pus, the same obtained throughout their whole extent, between the cruræus and vasti, and in the structure of the muscle itself. On dividing the deltoid, to ascertain the cause of the pain in the shoulder, pus was found between it and the capsule of the joint, which was quite healthy. On opening the cranium, the tunica arachnoides was found thickened and opaque, and "the cellular tissue, connecting the pia matral vessels, was found completely saturated with serous fluid." On tearing this up from the sulci, the convolutions seemed shrunk and shrivelled. The divided cerebral substance was very vascular, covered with bloody points, and blood exuded on pressure. The lateral ventricles contained no fluid, it having passed through the third and fourth ventricles into the vertebral canal. The spinal marrow itself could not be examined.

3. *Nævus Maternus.* An infant, four months old, was admitted, with subcutaneous nævus on the middle of the dorsal spine, an inch in diameter. At birth, it was about the size of a sixpence. When received, the nævus resembled half a small orange, in size and form. It had a spongy feel, and was reducible by pressure to nearly half its natural size. Mr. Lawrence passed a needle and double ligature through the base of the tumour, and then, separating the threads, drew them round, so as to encompass the whole base. The child cried incessantly during the remainder of the day, and was convulsed in the night. Next day it was fretful, but took the breast towards evening. It was convulsed the second night. *3d day.* Some bleeding occurred, but was suppressed by cold applications. *4th day.* Child very ill. Mr. L. sliced off two-thirds of the tumour, and then applied lint, moistened with cold water. *5th day.* The infant much better, and takes the breast. *6th day.* The ligatures removed, a poultice applied; and, on the 7th day, the tumour sloughed. The ulcer granulated kindly, and the child left the hospital cured.

4. *Dislocation of the 4th Cervical Vertebra, without Fracture.* A stout young man was brought into Bartholomew's, on the 8th January, under Mr. Lawrence. He had fallen with a barrel on his shoulder, while descending some steps, and the present accident was the result. He has now complete paralysis (of sensation and motion) in all parts below the neck. The respiration is performed solely by the diaphragm—he is sensible—pulse weak and slow—surface cold—priapism. After some hours, re-action took place, and venesection was performed, while purgatives were administered. Urine drawn off by the catheter. *9th.* A little sensibility in the front part of the chest, and some motility in the arms. He died on the 11th.

*Dissection.* There was complete dislocation of the 4th cervical vertebra from the fifth. Its inferior oblique articulating surfaces had passed in front of the superior articulating surfaces of the fifth cervical vertebra—its body, separated from the intervertebral substance, stood over that of the fifth, by its whole depth. No fracture could be discovered. The viscera were sound. It is to be regretted that no account is given of the state of the spinal medulla in this rare case. No doubt it was completely compressed.

5. *Cancer of the Penis.* We notice this case (Lancet, No. 191) to shew the influence of diet on foul sores, as well as on the constitution. A man presented himself to Mr. Lawrence, with unequivocal cancer of the penis, in an advanced stage. Three-fourths of the glans had been destroyed, and the sore had a sloughy, phagedenic aspect, with thick, hard, everted edges, and sanious fetid discharge. The glands in the groin were also hard and enlarged. The countenance was anxious, the pulse quick and small—the tongue loaded—bowels constipated. This man, who had a wife and five children to support, was ordered by the surgeon who attended him to take "*at least half a dozen of glasses of Madeira and a pound of beef-steaks daily.*" The consequence of this was, an evident deterioration of his health, which had previously continued uninfluenced by the local complaint. Mr. Lawrence very judicously advised him (as he could not come into the hospital) "to discontinue his fattening treatment—to substitute a light, unirritating diet, such as gruel, beef-tea, &c.—and to interdict himself from all vinous and fermented liquors." This regimen, with a bread poultice, and some aperient medicine, soon shewed an evident effect on the constitution, and even the sore. In ten or fourteen days the patient again presented himself. The ulcer had lost its sloughy aspect—it had a clear and granulated appearance, the discharge being diminished, and less fetid. The pain had also greatly diminished—the tongue was cleaner—and the countenance more healthy. The local complaint had gone past the period for an operation; but the malady was likely to be much mitigated by the system of diet directed by Mr. Lawrence.

6. *Erysipelas treated by Incisions.* T. R. aged 53, fell, and struck his elbow on the pavement, three days before admission, (Nov. 29th.) This was followed by an inflammatory swelling, distinctly circumscribed, extending from the olecranon, along the outer side of the fore-arm. There is a small laceration of the integuments over the olecranon, extending into the bursa, with discharge of some serous synovial fluid. The general health undisturbed. Calomel and jalap—senna and salts. Inflammation rose, and required venesection. On the 1st December, there was general inflammation, bearing the character of phlegmonous erysipelas, throughout the whole extremity below the elbow. Mr. Lawrence made two incisions with the scalpel, through the skin and cellular tissue, in the whole extent of the inflamed part—from above the elbow to near the wrist. The exposed cellular membrane was inflamed and thickened, and, in some places, was loaded with yellow and dusky effusion. 2d. The cellular tissue, and even the skin, is sloughing in some points—much constitutional irritation. *Purgation.* 3d Is much better—inflammation subsiding. The case, after this, went on favourably, and the man was discharged cured on the 17th Dec.—*No.* 186.

7 *Syphilis communicated to a Fœtus.* A married female had four healthy children, after which she contracted a syphilitic sore, of which she was cured in the hospital. The two next children were still-born—and the third was the patient now under consideration, born four years after the cure of the chancre above-mentioned. In three days after its birth, red patches were seen about the umbilicus—and, in the course of another week, on the palpebræ, with desquammation of cuticle on the hands and feet. The child was brought to the hospital, got some powders, and seemed cured in a short time. Three months afterwards it was brought back, with copper-coloured blotches about the head and face, followed by excoriations about the anus—thickening, redness and excoriation of the palpebral margins—yellow, viscid discharge from the nostrils—thickening of the alæ nasi—a circular sore, the size of a shilling, on the perineum, with raised, but smooth surface. Four grains of the hydrarg. cum creta night and morning, for three weeks, nearly dissipated all these symptoms; but one powder daily, for some time longer, was recommended.

We think it very doubtful that this female's word could be relied upon, as to the four years' immunity from syphilis previous to the birth of this last child.—*No.* 186.

8. *Phagedenic Ulceration.* A man was admitted under Mr. Lawrence, presenting a phagedenic ulcer, that had almost entirely destroyed the glans penis, and still forming a deep foul excavation, through which the urine was discharged. There was also superficial ulceration of the tonsils, and a foul ulcer on the side of the pharynx. Mr. L. considered the disease to be *phagedenic venereal*, the patient having had a gonorrhœa five months previously, and also a sore on the glans, for which no active remedies had been employed. Mr. L. determined not to use mercury internally, but only cinnabar fumigations, poultices, conium, and milk diet. Afterwards some wine was allowed, as several hæmorrhages had occurred. This treatment was pursued, with little variation, for seven weeks, when the ulcer of the penis was healed, as also were those of the throat. In another fortnight, the man was discharged from the hospital cured.—*No.* 186.

9. *Lepra Alphoides.* A little girl, aged ten years, was admitted with this eruption, which had existed for four months. The trunk, extremities, and hairy scalp, are studded with small, whitish, circular scales, unattended by any uneasy sensation, except slight itching when warm in bed. Mr. Lawrence ordered the warm bath thrice a week, with five grains of hyd. c. creta every second night, with some rhubarb the succeeding morning. This plan made little impression on the complaint, till salivation happened to take place, when the scales began to decline, and in three weeks she was discharged cured.—*No.* 187.

10. *Impetigo Figurata.* A case of this kind, for the description of which we refer to Bateman, Willan, Plumbe, or ony other writer on cutaneous diseases, is reported from Mr. Earle's wards. The patient was a young man of 19 years of age, who had had this eruption for 12 months, on the lower part of one leg, and on part of both hands. He was otherwise healthy, but when the pustules break and discharge, they occasion a troublesome itching, heat, and smarting. Mr. Earle ordered a lotion composed of four drachms of sulphuret of potass to a pint of distilled water—five grains of blue pill every night—and a pint of the decoct. sarsæ daily. Under this treatment considerable amendment ensued, but it does not appear that the patient was entirely cured.—*No.* 188.

11. *Phlebitis.* Another fatal case of this kind is recorded in Lan. No. 192, from Bartholomew's. The patient, a man aged 47, applied for relief of thoracic inflammation (Feb. 27th) and was bled. In three days he returned, complaining of pain in the wound, with inflammation of the surrounding cellular tissue, and evidently labouring under pulmonic inflammation. He was taken into the hospital—thirty leeches were applied to the inflamed arm, with a poultice—and antimonials in large doses (℥ss. of the liq. ant. tart.) given every four hours. Four doses, or grains of the tartrite were taken, without any sensible effect. The fever and inflammation (thoracic) running high, he was bled to 20 ounces, and had calomel and jalap, after which the *antimonial* as before. We need not pursue the treatment, which was simply antiphlogistic. The pulmonic inflammation was removed, or nearly so; but another and more dangerous phlogosis was strongly developing itself.

The arm (7th day) was now swelled—pus was discharged from the orifice when pressure was made over the track of the vein—diarrhœa—rigors. The antimony, calomel, &c. were discontinued. His debility and irritability now became considerable, and he lingered under irritative fever till the 31st March, when he expired, having discharged purulent expectoration for several days, and evinced high delirium prior to dissolution.

*Dissection.* The median and basilic veins were impervious, and reduced to a cord-like substance. The basilic vein led into an abscess, two inches above the original wound, situated in the track of the same vessel. Above the abscess, the basilic vein was lined with false membrane, which blocked up its calibre. There were several white spots on the surface of the heart, and extensive adhesions between the pleura costalis and lungs. In the lungs there were several vomicæ formed—the bronchia filled with mucus—abdominal viscera healthy—arachnoid over the hemispheres thickened and opake—pia mater infiltrated—and some ounces of yellow serum in the lateral ventricles.

In this case there was evidently a renewal of the pulmonic inflammation, when the fever of phlebitis became established—thus rendering the disease much more dangerous and intractable than simple phlebitis.

### II.

### GUY'S HOSPITAL.

1. *Scrotal Hernia—Unsuccessful Termination.** ———, æt. 62, entered Guy's, Feb. 13th, under Mr. B. Cooper, with a scrotal hernia which he had had for twenty years, but which had descended ten days previously, and could not be reduced by his medical attendant. On his admission he had the usual symptoms of strangulated hernia, and after the taxis, &c. had been ineffectually employed, the operation was performed. On opening the sac a large portion of omentum was seen, which adhered to it and to a small knuckle of intestine which lay beneath. As its vitality was much impaired, it was removed, save a small portion at the neck of the sac. The intestine was "dark coloured, and dull in appearance." "The stricture which was at the internal ring, was freely divided," and the intestine easily returned. In about three quarters of an hour, symptoms of collapse took place, with vomiting of feculent matter, and in six hours the patient was dead.

*Dissection.* The omentum in the sac was gangrenous; on opening the abdomen, the pelvis was found filled with feculent matter, and lying near the mouth of the sac was a fold of the ileum, of a dark colour, and evidently the knuckle of intestine which had been strangulated, and returned. By squeezing the intestinal contents they were seen to pass out of two small openings in the gut, one on each side, just where the stricture had been. These openings looked like ulcers, and the peritoneal coat appeared to be destroyed in several parts about the seat of stricture.

*Remarks.* After ten day's strangulation, there was no very great chance of the operation succeeding, but yet it was quite right to give the patient that chance. We are almost inclined to doubt the propriety of returning the gut in this case, for in these old adherent herniæ, experience we apprehend is against, rather than for the practice. Besides, if the report be correct, the gut " certainly seemed be closely approaching to, if not

* Lancet, No. 184, 1827.

actually in a state of mortification." At the same time these appearances of "approach to mortification" in strangulated intestine are very deceptive.

2. *Fistula in Perineo.*† G. B. æt. 50, entered Guy's Hospital, January 10th, under Mr. Morgan, with fistula in perineo.

The treatment consisted in first passing a catheter, by which the canal of the urethra was found to be very little obstructed. The fistula was immediately behind the scrotum and tortuous in its course; the integuments around much thickened. The sinus was freely laid open with a bistoury, apparently to its termination in the urethra, and a gum catheter introduced. This was to be worn constantly. In spite of this, the urine, at the end of three weeks, continued to dribble from the wound, and though this had nearly closed externally, Mr. Morgan suspected that the irritation of the catheter might prevent the healing process within, he accordingly withdrew the instrument. This had no effect, and the parts were divided a second time, the wound dressed with lint, and the gum catheter left in the bladder. The patient after a certain time left the hospital quite cured.

3. *Spinal Injury.* Harriet Cornix, æt. 17, was admitted, March 14th, under the care of Mr. Morgan. Six months previously, whilst getting out of a stage-coach, she slipped and fell upon her left side on the pavement, striking her head at the same time. Whilst falling, she felt something "push her neck aside," and on recovering her senses, for she was stunned for upwards of half an hour, she found her neck immoveably flexed towards the left side, the vision of both eyes was dim, that of the left continued so for several hours, the right eye recovered itself sooner. A surgeon applied leeches to the occiput, and, in the course of ten days, the patient complained only of the distortion. After a time she began to feel a tingling pain in the neck and left arm, extending down the spine to the loins. She consulted several medical men, Mr. Stanley, Mr. White, Dr. Whiting, &c. but obtained no relief. On admission the neck was found permanently twisted to the left side, the least attempt at motion producing extreme pain. She had various nervous symptoms, as pain on raising the left arm—occasional tremors of the whole frame, with spasmodic twitchings of the muscles of the lower jaw and left side of the face—the tremors too, on close examination, were seen to be confined to the muscles of the left side of the body, and pressure on the spines of the cervical or upper dorsal vertebræ produced these twitchings, and a violent darting pain through the body. This last was at times present, independent of pressure. "On accurately tracing the spinous processes, we could not discover any displacement; *this was the opinion also of Mr. Morgan!*" There was lateral curvature, the convexity towards the right side—health good—bowels and catamenia regular.

Twelve ounces of blood were taken from the back of the neck by cupping, and an issue on each side of the lower cervical vertebræ—half a drachm of carbonate of iron thrice a day. Subsequently half an ounce of the decoction of aloes, with two drachms of the compound spirit of ammonia and peppermint water were given thrice a day—the dose of the decoction afterwards reduced to two drachms. Calomel and scammony every second night, and moxa over the lower cervical spinous processes. The carbonate of iron was omitted in the course of a few days. The patient at the date of report, April 14th, was much as when she entered the hospital.

4. *Fatal Spinal Disease.*—T. Y. æt. 22, in February, 1826, fell from a cart,

---

† Lancet, No. 189, 1827.

but was able to walk home, and follow his usual employment. He suffered, however, occasionally violent pain in the back, and distressing weakness, which increased so as to confine him to his bed. He was bled and blistered, and had what he called convulsion in his lower limbs, with great loss of power. He went for two months to the Margate Infirmary. On his admission, Feb. 21st, 1827, into Guy's, volition and sensation were entirely gone in both legs below the knee, and they were imperfect in the thighs. He had constant gnawing pain in the back, which prevented him from sleeping; there was some slight projection at the lower dorsal vertebræ, but no tenderness on pressure. There was incontinence of urine, and when the bowels were much relaxed, the fæces passed involuntarily. In spite of blisterings and cupping, the man became more feeble—sloughs formed on the nates, and in six weeks from the time of his entering the hospital he died. No dissection was allowed.

This case is interesting. In the absence of direct proof by dissection, it seems highly probable, that chronic inflammation was set up in the medulla or its membranes, in consequence of the injury, and that this inflammation terminated in alteration of structure.

5. *Nævi Materni.* The profession was favoured some years ago with a paper from Mr. Wardrop, published in the Medico-Chirurgical Transactions, on the subject of a natural cure which nævi materni sometimes undergo, by a process of spontaneous ulceration. The distinguished surgeon abovementioned imitated this natural process, by applying a strong solution of oxymuriate of mercury to a nævus on a child's back. In this instance, the skin ulcerated rapidly, destroying the substance of the tumour Two cases are reported from Panton Square, in No. 181 of the Lancet, where a similar practice was adopted.

*Case* 1. A child, aged 13 months, had a small nævus, the size of a sixpence, on the middle of the frontal region, the central portion of which was of a bright red colour, and to this centre several tortuous vessels ran from the circumference. A piece of adhesive plaster, with a hole in its middle, was applied over the nævus. Through this aperture the kali purum was admitted to the surface. An eschar formed and separated, and subsequent ulceration destroyed the whole of the nævus. The part was cicatrized in three weeks.

*Case* 2. This was nearly similar, except that the nævus was situated on the cheek. The same treatment was adopted, and the same result ensued.

We lately saw a nævus situated on a child's head, and projecting a couple of lines in height, being about the size of a shilling. By some accident the nævus was irritated—then inflamed, ulcerated, and became destroyed. The child appeared to suffer a good deal during this ulcerative process.

6. *Spina Bifida cured.* Mr. Probart, of Hawarden, has communicated a case of spina bifida cured by repeated punctures and discharge of fluid from the tumour. There was a loss of bony substance in the situation of the second lumbar vertebra, and the lower extremities were completely paralytic. When the child was about three months old, the operations were commenced. After a few puncturings, there came on considerable inflammation in the integuments of the tumour, and the child had some smart convulsive paroxysms. But, by emptying the bowels, and applying leeches to the parts, the inflammation was reduced, and the convulsions ceased. The integuments covering the spina bifida became thickened, and then a plaster and compress were applied with pressure. By these means the tumour was reduced, and ultimately disappeared, leaving a depression in its place.—*Lancet, No.* 186.

